# VOPP1 promotes breast tumorigenesis by interacting with the tumor suppressor WWOX

**DOI:** 10.1186/s12915-018-0576-6

**Published:** 2018-10-02

**Authors:** Florian Bonin, Karim Taouis, Paula Azorin, Ambre Petitalot, Zakia Tariq, Sebastien Nola, Nadège Bouteille, Sandrine Tury, Sophie Vacher, Ivan Bièche, Khadija Ait Rais, Gaelle Pierron, Laetitia Fuhrmann, Anne Vincent-Salomon, Etienne Formstecher, Jacques Camonis, Rosette Lidereau, François Lallemand, Keltouma Driouch

**Affiliations:** 1 Pharmacogenomics Unit, Department of Genetics, Institut Curie, 75005 Paris, France; 2 Present address: INSERM U950, Institut Jacques Monod, Université Paris Diderot, Sorbonne Paris Cité, 75013 Paris, France; 3 Somatic Genetics Unit, Department of Genetics, Institut Curie, 75005 Paris, France; 4 Pathology, Department of Tumor Biology, Institut Curie, 75005 Paris, France; 5 Hybrigenics Services, 75014 Paris, France; 6 INSERM U830, Institut Curie, 75005 Paris, France

**Keywords:** VOPP1, WWOX, Breast tumors

## Abstract

**Background:**

The WW domain-containing oxidoreductase (*WWOX*) gene, frequently altered in breast cancer, encodes a tumor suppressor whose function is mediated through its interactions with cancer-related proteins, such as the pro-apoptotic protein p73α.

**Results:**

To better understand the involvement of WWOX in breast tumorigenesis, we performed a yeast two-hybrid screen and co-immunoprecipitation assays to identify novel partners of this protein. We characterized the vesicular overexpressed in cancer pro-survival protein 1 (VOPP1) as a new regulator of WWOX. In breast cancer cells, VOPP1 sequestrates WWOX in lysosomes, impairs its ability to associate with p73α, and inhibits WWOX-dependent apoptosis. Overexpressed VOPP1 potentiates cellular transformation and enhances the growth of transplanted tumors in vivo. VOPP1 is overexpressed in breast tumors, especially in tumors that retain WWOX. Moreover, increased expression of VOPP1 is associated with reduced survival of patients with WWOX-positive, but not with WWOX-negative, tumors.

**Conclusions:**

These findings emphasize the importance of the sequestration of WWOX by VOPP1 in addition to WWOX loss in breast tumors and define *VOPP1* as a novel oncogene promoting breast carcinogenesis by inhibiting the anti-tumoral effect of WWOX.

**Electronic supplementary material:**

The online version of this article (10.1186/s12915-018-0576-6) contains supplementary material, which is available to authorized users.

## Background

*WWOX* has been characterized primarily as a tumor suppressor gene spanning the fragile site FRA16D [1]. In the last few years, it has become evident that WWOX protein has pleiotropic functions, playing critical roles not only in cancer but also in other severe human pathologies including metabolic disorders and CNS-related syndromes [2, 3]. Importantly, homozygous mutations of *WWOX* were recently identified as responsible for an inherited neural disorder characterized by cerebellar ataxia, epilepsy, and mental retardation (OMIM **#** 616211) [4].

In human cancers, according to The Cancer Genome Atlas, mutations affecting the *WWOX* gene are rare events (Reference 3 and http/www.cbioportal.org for updates). However, altered *WWOX* expression is frequent in tumors, which is mainly due to hemi- and homozygous loss and rearrangements on chromosome arm 16q [3, 5]. Heterozygous deletions of *WWOX* gene were observed in more than 50% of breast tumors and reached up to 80% in ovarian carcinomas [3, 5–7]. In addition, loss or reduced WWOX expression may also result from epigenetic alterations and post-translational modifications such as promoter hypermethylation, miRNA targeting, and proteasomal degradation [5, 8, 9]. Overall, low levels of *WWOX* expression were associated with a poor clinical outcome, which suggested an important role of the protein in a broad range of human cancers [5].

The tumor suppressor function of *WWOX* was demonstrated by studies showing that its ectopic overexpression inhibited tumor growth [10–13]. In genetically engineered mice, complete ablation of *WWOX* resulted in early postnatal lethality, which precluded the analysis of *WWOX* involvement in tumorigenesis in *WWOX*−/− mice. However, increased incidence of spontaneous and induced tumors of the lung, breast, and B cell lymphomas was observed in *WWOX* hypomorphic and heterozygous *WWOX*+/− animals [14–17].

The WWOX protein consists of two N-terminal WW domains (WW1 and WW2) and a C-terminal short-chain dehydrogenase/reductase domain (SDR) [6, 18]. The WW domains are well-characterized modules that mediate protein-protein interactions [19]. The WW1 domain is the predominant functional interacting domain of WWOX, which belongs to the largest group of WW domains, i.e., group I that specifically recognizes proteins with PPxY consensus motifs [19]. A recent report revealed that the WW1 module interacts with an extended list of L/PPxY-containing proteins, thereby acting as a versatile platform linking WWOX with numerous multiprotein complexes involved in important processes such as transcription, RNA processing and splicing, chromatin remodeling, metabolism, and various cancer-related pathways [20]. Thus, WWOX suppressor activity relies on its binding capacities to known cancer-related proteins such as p73, deltaNp63, AP2γ, c-jun, Erbb4, c-Met, RUNX2, and Dvl-2 [21]. Several of these WWOX-binding proteins are transcription factors or co-factors. In most cases, WWOX suppressed their activity by sequestering them in the cytoplasm [3, 21, 22].

At the cellular level, WWOX has been shown to promote apoptosis. Restoration of WWOX expression in several types of cancer cells induced apoptosis [11, 12]. In particular, Aqeilan et al. demonstrated that WWOX bound to and sequestered p73 in the cytoplasm. The cytoplasmic WWOX-p73 complex induced apoptosis independently of p73 transcriptional activity [23]. More recently, emerging evidence also suggests an important role for WWOX in the DNA damage response (DDR). It was shown that DNA damage triggered WWOX accumulation in the nucleus where it bounds to ATM protein and activated DDR [24]. Therefore, loss of WWOX in human tumors might also lead to genomic instability, another hallmark of cancer [25].

In this report, we screened for new WWOX-interacting proteins to gain insight into the involvement of the WWOX tumor suppressor in breast cancer. We highlighted VOPP1 as a new molecular partner of WWOX. The *VOPP1* gene (previously known as *ECOP* or *GASP*) is localized at the frequently amplified 7p11.2 locus and is often co-amplified with *EGFR* [26, 27]. *VOPP1* overexpression has been observed in multiple malignancies such as glioblastoma and gastric, head and neck, lung, and breast cancers [28–30]. Interestingly, in different cellular models, *VOPP1* depletion resulted in apoptosis, while ectopic expression conferred a pro-survival phenotype to cancer cells, which strongly suggested VOPP1 as an anti-apoptotic protein [31, 32]. Our study aimed at better characterizing the role of WWOX and VOPP1 binding in breast cancer.

## Results

### VOPP1 interacts with WWOX tumor suppressor

We performed a yeast two-hybrid screen to identify new WWOX-interacting proteins [22, 33]. As a bait, we used the full-length protein (NP_057457) and a shorter isoform (WWOX_v2_, NP_570607) containing the two WW domains and a truncated SDR domain. We screened at saturation a highly complex human placenta library and identified ten clones encoding the C-terminus region of the VOPP1 protein.

**Fig. 1:**
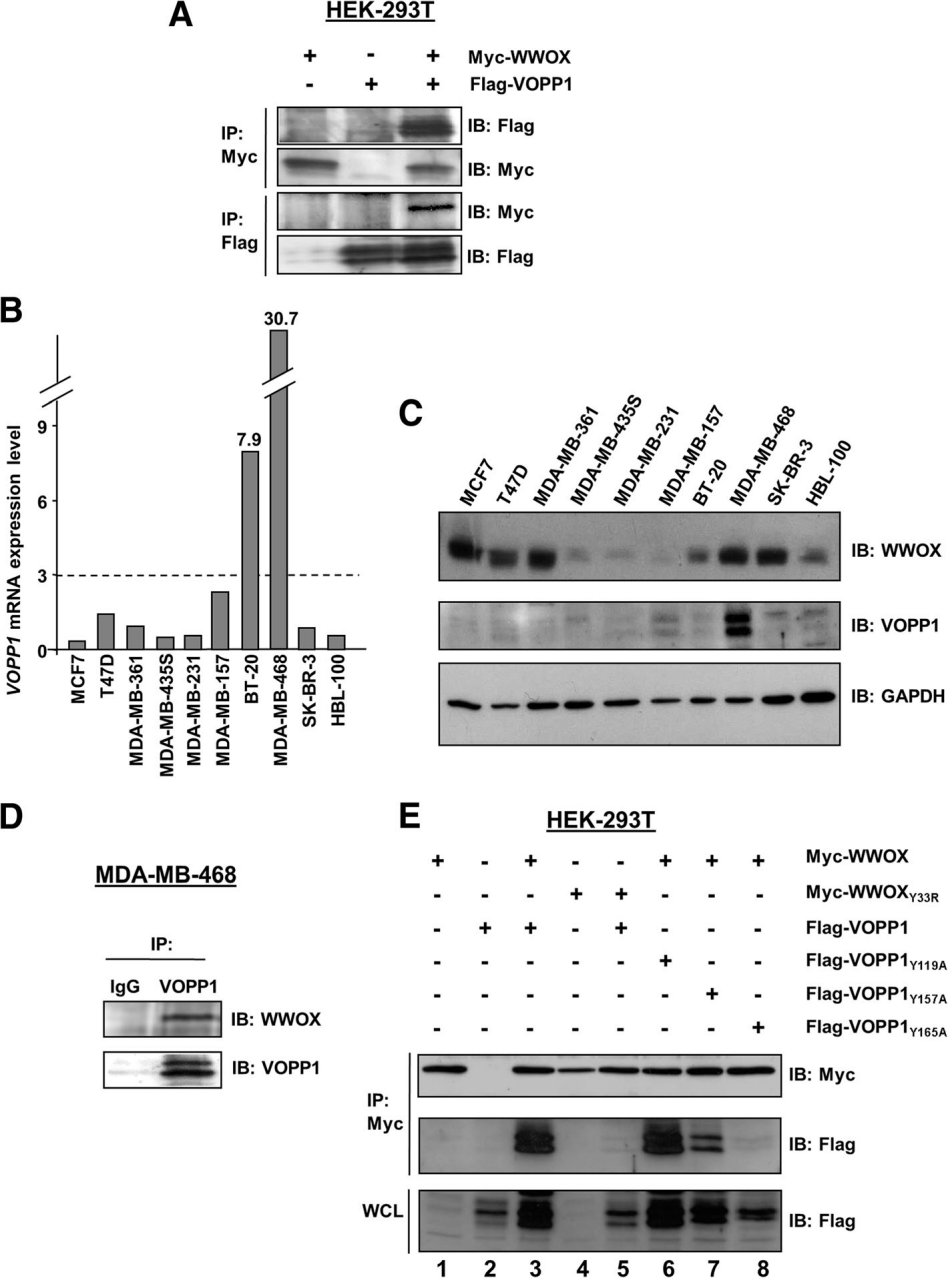
WWOX and VOPP1 expression, interaction, and binding domains. **a** Co-immunoprecipitation of Myc-WWOX and Flag-VOPP1 in HEK-293T cells. Antibodies for immunoprecipitation (IP) and immunoblotting (IB) analyses are indicated. *VOPP1* mRNA (**b**) and protein (**c**) expression in ten breast cancer cell lines. **d** Co-immunoprecipitation of the endogenous WWOX/VOPP1 complex. MDA-MB-468 cells were immunoprecipitated with either normal rabbit IgG as a negative control (IgG) or anti-VOPP1 antibody (VOPP1). Immunoprecipitates were examined by immunoblotting with anti-WWOX antibody. **e** Co-immunoprecipitation with wild-type and mutants forms of WWOX and VOPP1 constructs WWOX_Y33R_, VOPP1_Y119A_, VOPP1_Y157A_, and VOPP1_Y165_ were performed as indicated in HEK-293T cells. WCL, whole cell lysate

To validate that the VOPP1-WWOX interaction occurred in mammalian cells, we generated a Flag-tagged VOPP1 expression construct that was co-expressed with a Myc-tagged WWOX plasmid in HEK-293T cells. Cell lysates were immunoprecipitated with anti-Flag or anti-Myc antibodies. Flag-VOPP1 was specifically co-immunoprecipitated with Myc-WWOX by an anti-Myc antibody and reciprocally (Fig. 1a).

To examine the existence of endogenous VOPP1-WWOX complexes in breast cancer cells, we first evaluated the expression of *VOPP1* transcripts and proteins in a series of ten human breast cancer cell lines (Fig. 1b, c). The highest levels of expression for VOPP1 were observed in MDA-MB-468 cells. This line was thus used to perform a co-immunoprecipitation assay using an anti-VOPP1 antibody (Fig. 1d). We found that WWOX specifically co-precipitated with VOPP1 indicating that endogenous WWOX and VOPP1 were physically associated in breast cancer cells.

Next, we investigated which domains of both proteins were crucial for WWOX-VOPP1 interaction (Fig. 1e). First, we investigated the binding capacity of WW1, the predominant interacting domain of WWOX [20]. For that, we generated the WWOX_Y33R_ mutant and tested its ability to bind VOPP1, the Y33R mutation inhibiting the binding ability of WW1 [23]. The Y33R mutation in WWOX abolished the WWOX-VOPP1 interaction (Fig. 1e), which indicated that the WW1 module was indispensable for the WWOX-VOPP1 association. Then, by examining the VOPP1 protein sequence, we identified three PPxY motifs in the proline-rich C-terminal region of the protein: PPYY_119_, PPAY_157_, and PPPY_165_. We generated three VOPP1_PPxY_ mutants in which the conserved tyrosines were replaced by alanine residues and tested their ability to bind WWOX. The results showed that WWOX-VOPP1 interaction was strongly affected by the mutation of tyrosine 165 (Fig. 1e). These observations indicated that the PPPY_165_ motif was required to sustain a robust interaction of VOPP1 with the WW1 domain of WWOX. Altogether, these results indicated VOPP1 as a novel partner of WWOX.

### VOPP1 sequesters WWOX in the lysosomal vesicles

**Fig. 2:**
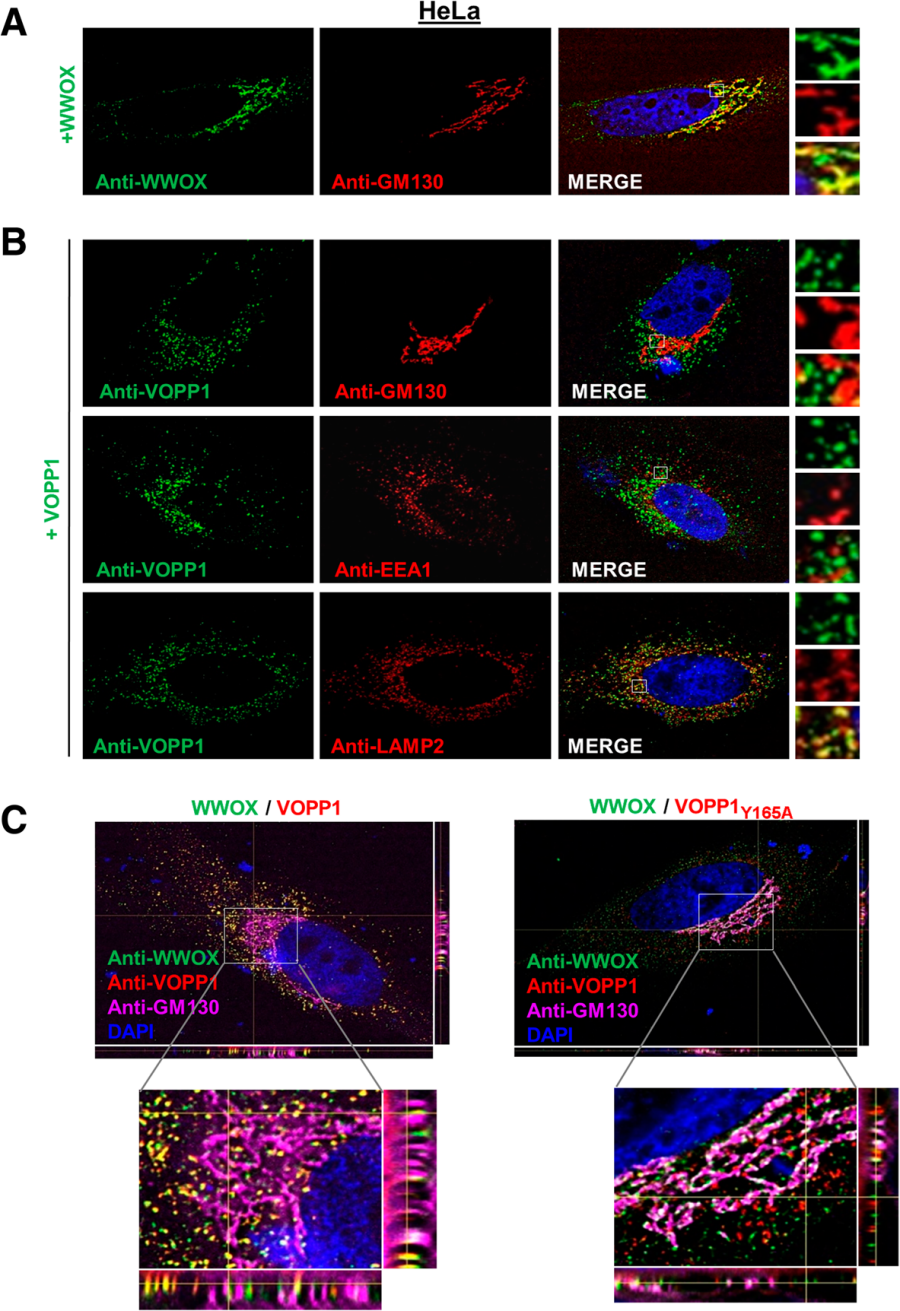
Subcellular localization of WWOX, VOPP1, and WWOX/VOPP1 complex in Hela cells. **a** Cells were transfected with the WWOX expression vector. The overlay of immunostaining with anti-WWOX and anti-GM130 antibodies indicates a localization of WWOX protein in the Golgi apparatus. **b** Cells were transfected with VOPP1. VOPP1 labeling coincides with LAMP2 patterns but not with GM130 or EE1A distribution suggesting lysosomal localization of VOPP1 protein. **c** Hela cells were transfected with WWOX and VOPP1 (left panels), or WWOX and VOPP1_Y165A_ (right panels) expression vectors as indicated. After immunostaining with anti-VOPP1, anti-WWOX, and anti-GM130, cells were counterstained with DAPI. WWOX and VOPP1 colocalization was observed (yellow) in cells expressing VOPP1, but not VOPP1_Y165A_. All experiments were performed using 3D deconvolution microscopy and digital overlay of images (original magnification, × 100)

WWOX- and VOPP1-interacting proteins should reside, at least transiently, in the same cellular compartment. Human WWOX was reported to localize in the Golgi apparatus [10, 19], while VOPP1 displayed a vesicular distribution consistent with endosomal/lysosomal compartmentalization [28, 30]. To determine the subcellular localization of the WWOX-VOPP1 complex, we transiently expressed WWOX and VOPP1 either alone or in combination in HeLa cells. As expected, WWOX expressed alone was largely accumulated in GM130-positive structures in line with a Golgi localization [34] while VOPP1 expressed alone showed a close apposition to LAMP2 patches, but not to EEA1 (marker of early endosomes) or GM130 patches, indicating a late endosomal/lysosomal localization [35] (Fig. 2a, b). Interestingly, when co-expressed with VOPP1, WWOX completely lost its typical Golgi distribution and exhibited a punctate pattern consistent with lysosomal structures as shown by the staining overlap of WWOX-, VOPP1-, and LAMP2-positive vesicular bodies (Fig. 2c and Additional file 1: Figure S1A). We then asked whether the relocation of WWOX was related to the VOPP1 binding by co-expressing WWOX with the VOPP1_Y165A_ mutant. In VOPP1_Y165A_-expressing cells, WWOX largely remained in the Golgi compartment (Fig. 2c and Additional file 1: Figure S1B).

**Fig. 3:**
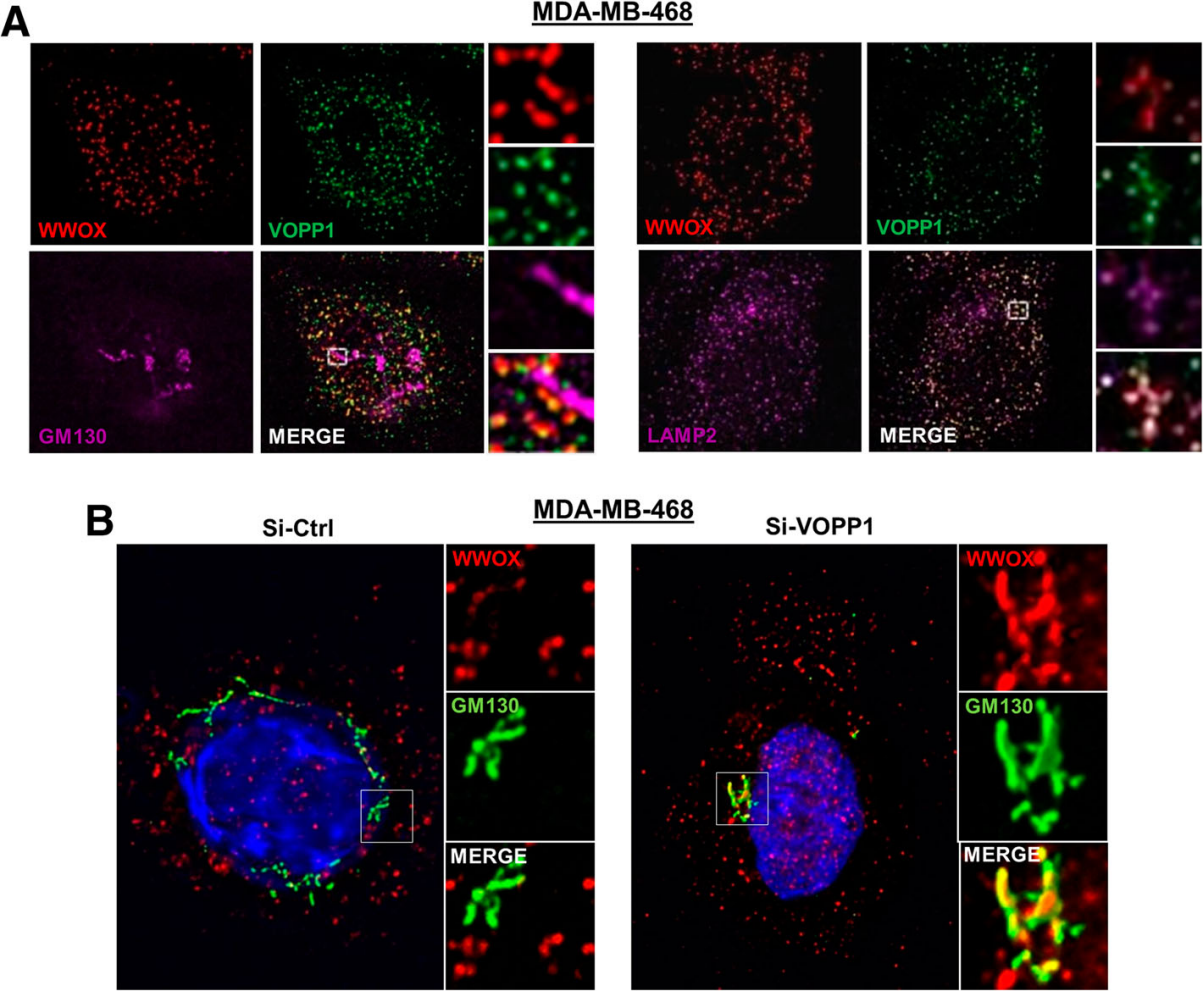
Depletion of VOPP1 induces subcellular relocalization of WWOX from the lysosomes to the Golgi apparatus. **a** Subcellular localization of endogenous WWOX and VOPP1 in MDA-MB-468. Cells were fixed, permeabilized, and immunostained with anti-WWOX, anti-VOPP1, and anti-GM130 (left panels), or anti-LAMP2 (right panels) antibodies as indicated followed by appropriated secondary fluor-conjugated antibodies. **b** The inhibition of VOPP1 expression induced the re-localization of WWOX in the Golgi apparatus. MDA-MB-468 cells were transfected with the control siRNA (Si-Ctrl) or *VOPP1* siRNA (Si-VOPP1) as indicated. Three days after the transfection, cells were fixed, permeabilized, and immunostained with anti-WWOX and anti-GM130 antibodies. Cells were then counterstained with DAPI and imaged using a fluorescence microscope (original magnification, × 100)

Next, we analyzed the localization of the endogenous WWOX-VOPP1 complex in MDA-MB-468 cells and observed no overlap of WWOX staining with GM130-positive structures (Fig. 3a). In contrast, perinuclear co-localization of WWOX, VOPP1, and LAMP-2 labeling was clearly distinguished, which indicated that the endogenous complex was associated with lysosomes (Fig. 3a). In these cells, we analyzed the effect of VOPP1 depletion on WWOX expression profile by RNA interference. In contrast to control cells (Si-Ctrl), WWOX extensively co-localized with the GM130-positive structures in si-*VOPP1*-treated cells (Si-*VOPP1*) (Fig. 3b). Thus, the inhibition of VOPP1 expression in MDA-MB-468 cells induced the re-localization of WWOX from the lysosomal vesicles to the Golgi apparatus.

Altogether, these results strongly indicated that the WWOX and VOPP1 complex resides in the late endosomes/lysosomes and VOPP1 had the ability to sequester WWOX in this subcellular compartment.

### VOPP1 inhibits WWOX-dependent apoptosis

**Fig. 4:**
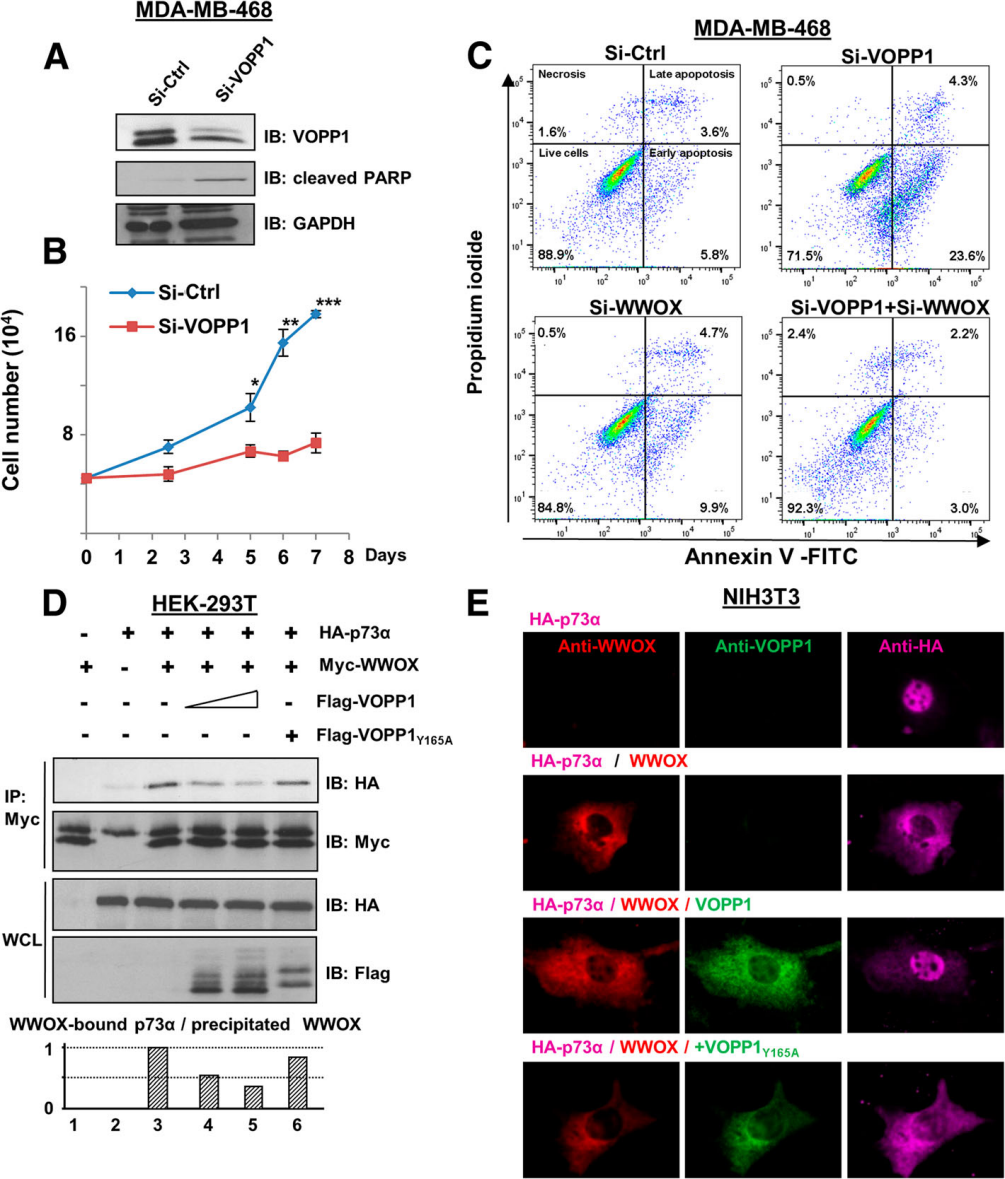
VOPP1 inhibits WWOX pro-apoptotic activity. **a** MD-MB-468 cells were transfected with control (Si-Ctrl) and *VOPP1* siRNAs (Si-VOPP1) for 3 days. Cellular extracts were immunoblotted with anti-VOPP1, anti-cleaved PARP, and anti-GAPDH (loading control) antibodies. **b** An MTS assay was performed at times as indicated; cell numbers are expressed as mean + SEM of triplicates from a representative experiment. Student *t* test (*p < 0.05; **p < 0.01; ***p < 0.001). **c** MD-MB-468 cells were transfected with control, *VOPP1*, and *WWOX* siRNAs as indicated. Three days later, cells were collected and the percentage of apoptotic cells was evaluated by flow cytometry. **d** HEK-293T cells were transfected with HA-p73α (0.5 μg), Myc-WWOX (0.5 μg), Flag-VOPP1 (0.5 or 1 μg), and Flag-VOPP1_Y165A_ (1 μg) expression vectors as indicated. Co-immunoprecipitation results show a decreased level of the WWOX-p73 complex in the presence of ectopic VOPP1 but not VOPP1_Y165A_. **e** Cells were transfected with HA-p73α alone or in combination with WWOX, WWOX and VOPP1, or WWOX and VOPP1_Y165A_ expression vectors as indicated. Twenty-four hours after transfection, cells were fixed, permeabilized, and immunostained with anti-WWOX, anti-VOPP1, and anti-HA antibodies followed by appropriate secondary fluor-conjugated antibodies. After immunostaining, cells were imaged with a fluorescence microscope and the images were overlaid (original magnification, × 100)

Our observations prompted us to investigate whether the VOPP1-WWOX interaction may have affected the apoptotic function of WWOX. First, we determined the effects of suppressing *VOPP1* on cell death in MDA-MB-468 cells. Si-RNA treatment (70% efficacy, Fig. 4a) reduced the viability of VOPP1-depleted cells compared to the Si-Ctrl-treated cells (Fig. 4b). Furthermore, VOPP1 silencing greatly increased the expression of cleaved PARP, a cellular marker of apoptosis (sevenfold increase, Fig. 4a). These results indicated that the depletion of VOPP1 in MDA-MB-468 cells affected cell viability by inducing apoptosis.

We then examined the ability of VOPP1 knockdown to induce the pro-apoptotic activity of WWOX. Upon Si-VOPP1 treatment, MDA-MB-468 cell death showed a fourfold increase (23.6% in Si-VOPP1 cells compared to 5.8% in control cells, Fig. 4c) as measured by Annexin V/PI staining coupled with flow cytometry. Remarkably, VOPP1 suppression had no effect in WWOX-depleted cells, which suggested specific regulation of WWOX-mediated apoptosis by VOPP1 protein (Fig. 4c). Conversely, WWOX-mediated cell death in A549 cancer cells was overcome by ectopic expression of VOPP1 but not VOPP1_Y165A_ indicating that VOPP1 overexpression inhibited WWOX-dependent apoptosis (Additional file 2: Figure S2A-B).

To highlight the underlying mechanisms, we examined the effect of VOPP1 on WWOX-p73α complex known to induce apoptosis [23]. By performing co-immunoprecipitation experiments, we found that ectopic expression of VOPP1, but not VOPP1_Y165A_, affected the ability of WWOX to associate with p73α (Fig. 4d). We also found that the sequestration of p73α in the cytoplasm by WWOX was inhibited by ectopic expression of VOPP1 but not VOPP1_Y165A_, which confirmed the negative effect of VOPP1 on the WWOX-p73α association (Fig. 4e). We then examined the marker of apoptosis, cleaved PARP, under these experimental conditions. We found that overexpressed WWOX increased cleaved PARP expression and that this effect was inhibited by ectopic expression of VOPP1 wild-type but not VOPP1_Y165A_ (Additional file 2: Figure S2C). However, we did not detect the significant cooperative effect of WWOX and p73α on cleaved PARP expression, probably due to the triple transfection condition. Indeed, in double transfection conditions, we found that WWOX and p73α strongly cooperate to induce cleaved PARP expression (Additional file 2: Figure S2C-D).

Altogether, these results suggested that VOPP1 inhibited WWOX-dependent apoptosis at least in part by preventing the WWOX-p73α interaction.

### VOPP1-mediated oncogenic properties and effect on tumorigenesis in vivo

**Fig. 5:**
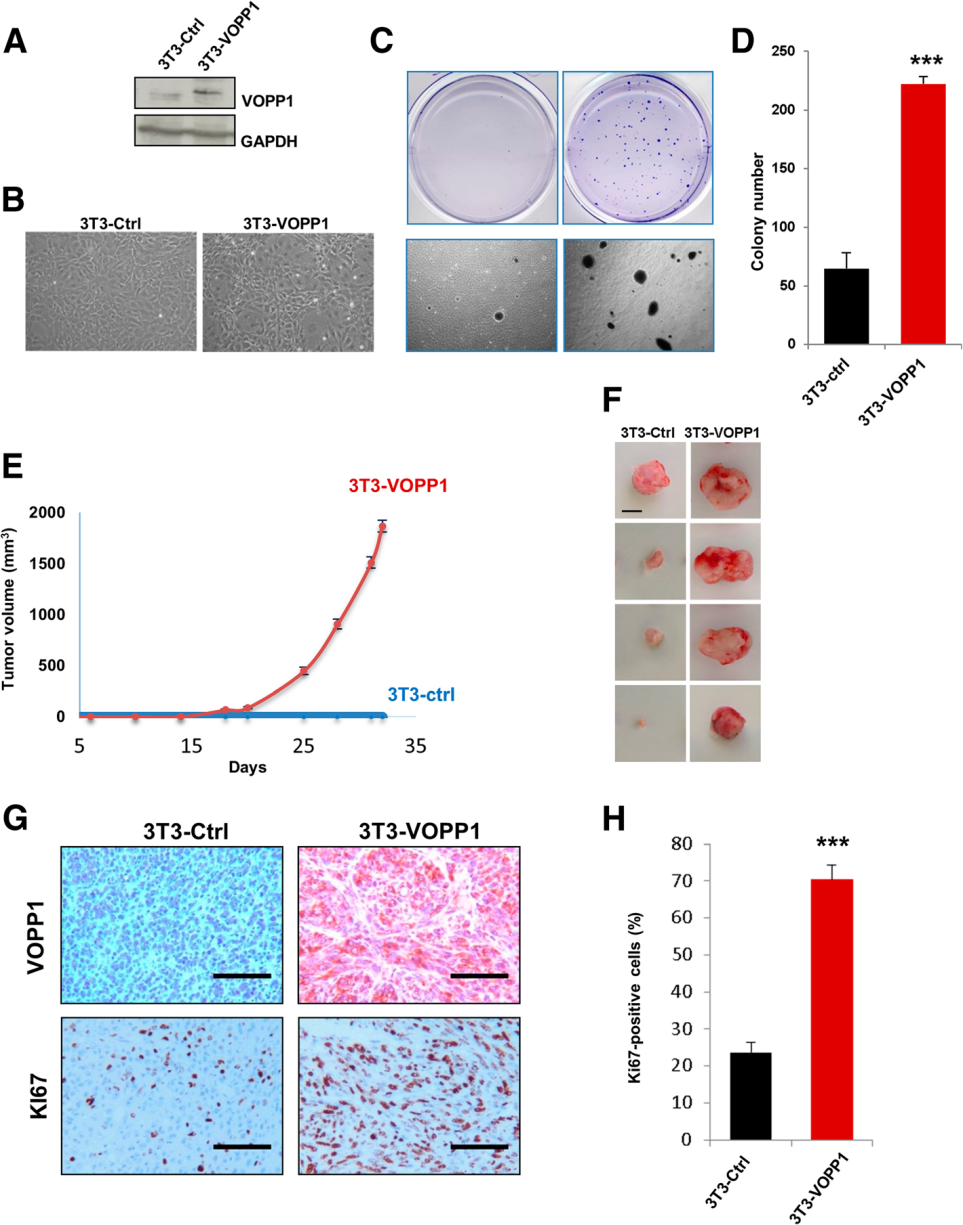
VOPP1 induces cell transformation in vitro and tumorigenesis in vivo. **a** NIH3T3 cells were stably transfected with control VOPP1-expressing vectors. Cellular extracts were immunoblotted with anti-VOPP1 and anti-GAPDH (loading control) antibodies. **b** Morphology of NIH3T3 cells stably expressing VOPP1 (3T3-VOPP1) or empty vector (3T3-ctrl). **c** Low-magnification photographs represent the results of soft agar colony formation assays showing the number and size of colonies formed by 21 days after plating 3T3-Ctrl and 3T3-VOPP1 cells. **d** The quantitative results of colony numbers are expressed as mean ± SEM of a representative experiment (*n* = 4). **e** 2.10^6^ 3T3-Ctrl and 3T3-VOPP1 cells were subcutaneously injected into the flanks of SCID mice, and tumor growth was measured over time (*n* = 10 per each group) up to 35 days. **f** 3T3-Ctrl and 3T3-VOPP1 mice were sacrificed when tumors reached a volume of approximately 2 cm^3^; otherwise, they were sacrificed at the end of the experiment (week 25, for 3T3-VOPP1 mice only), representative images of tumors (scale bar, 1 cm). **g** Immunohistochemical staining of 3T3-Ctrl and 3T3-VOPP1 tumors with indicated antibodies (scale bar = 100 μm). **h** Quantification of Ki67+ proliferating cells in tumors. Statistical analysis was made by performing the Student *t* test (*p < 0.05; **p < 0.01; ***p < 0.001)

Our findings led us to assume that VOPP1 might function as an oncogene. To test this hypothesis, we examined whether VOPP1 overexpression would be sufficient to transform non-cancerous cells. Stable expression of VOPP1 in NIH3T3 cells (3T3-VOPP1) resulted in classical morphological transformation as evidenced by the appearance of small, refractile, spindle-shaped cells (Fig. 5a, b). To directly assess the transforming potential of VOPP1, we performed a soft agar colony formation assay and found that stable expression of VOPP1 in NIH3T3 cells also induced anchorage-independent growth in soft agar as shown by an increase of both number and size of colonies compared to control cells (Fig. 5c, d). Furthermore, injection of 3T3-VOPP1 cells into immunocompromised mice led to tumor formation in vivo. The tumor growth rate and mean tumor volumes were drastically increased in mice injected with 3T3-VOPP1 cells compared to control cells (NIH3T3 cells stably transfected by the empty vector, 3T3-Ctrl) (Fig. 5e). Since no tumors were obtained in the control group at day 35, a second experiment was carried out and followed for a longer time period. At the end of the experiments (week 25), only half of the control mice had palpable tumors whereas all 3T3-VOPP1 mice were already sacrificed for tumors reaching the ethical size.

Immunohistological analyses of tumor xenografts showed a higher number of Ki67-positive cells in 3T3-VOPP1 tumors compared to the 3T3-Ctrl tumors (Fig. 5g, h).

Collectively, these results indicated that VOPP1 had transforming activity and induced a tumorigenic phenotype.

### High VOPP1 expression is associated with poor prognosis of breast cancer patients

**Fig. 6:**
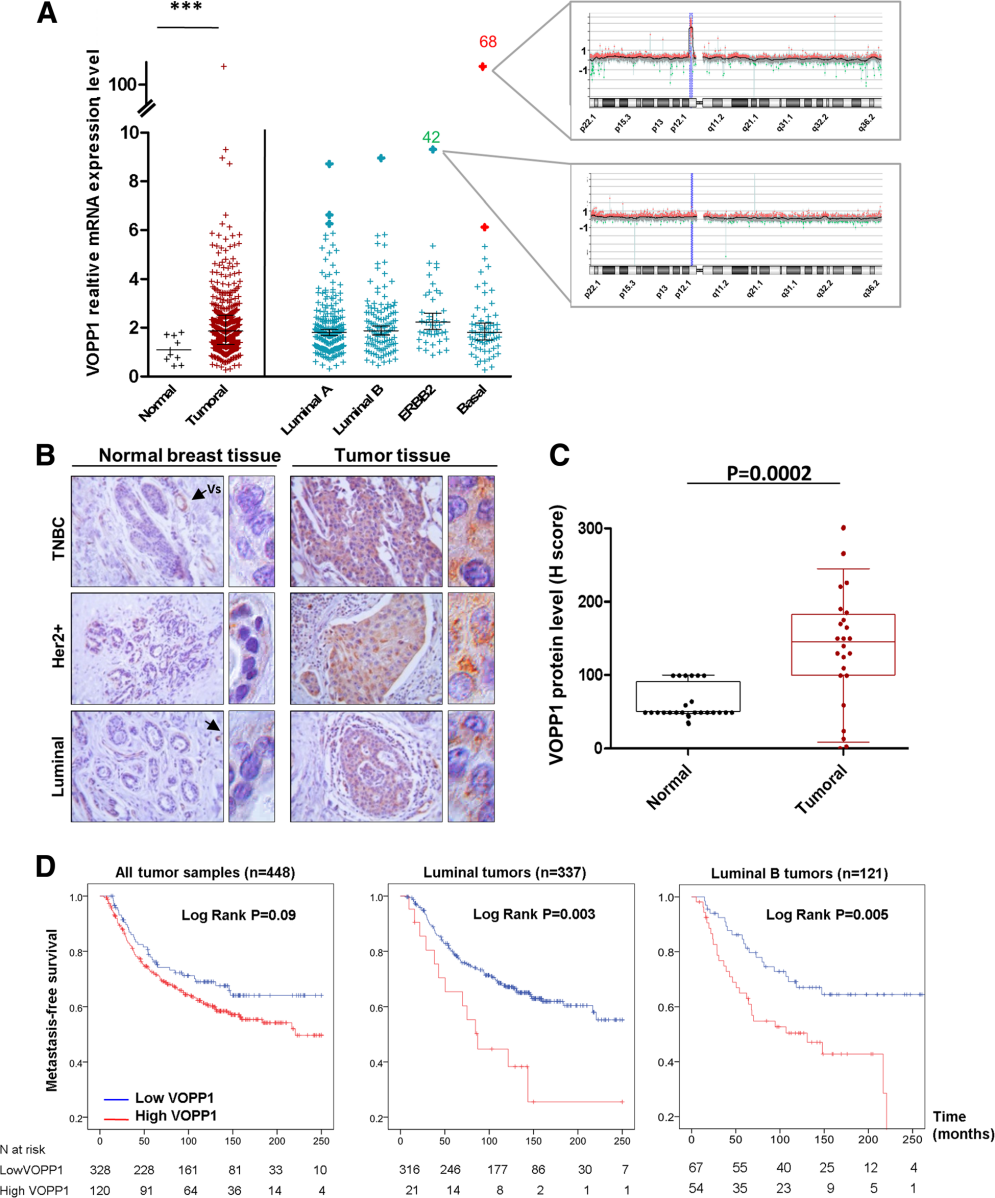
VOPP1 expression in human breast tumors. **a** Real-time PCR analysis of VOPP1 expression in 448 breast tumors. The CGH profiles showing the VOPP1 chromosomal region are highlighted for two tumors. Statistical analysis was performed using the Mann-Whitney *U* test (***p < 0.001). **b** Representative images of an immunohistochemical analysis of VOPP1 protein expression in a series of 24 tumors. Arrows indicate the blood vessels. **c** Quantification of the immunohistochemical results showed in **b**. **d** Kaplan-Meier curves showing metastasis-free survival rates of patients with tumors expressing high (red line) vs. low (blue line) levels of *VOPP1* mRNA

To evaluate the clinical relevance of VOPP1 in breast cancer patients, we investigated its expression in a series of 448 invasive tumors from breast cancer patients with known long-term outcome and ten normal breast tissues by qRT-PCR analysis (Additional file 3: Table S1). In line with studies on other tumor types [26, 28–32], we found that 25% of breast tumors (112/448) showed VOPP1 overexpression (≥ 2.5-fold increase as compared to expression in normal breast tissue) (Additional file 3: Table S1). Relative expression of VOPP1 was significantly increased in the primary breast tumors (*p* = 8.10^−4^, Mann-Whitney *U* test, Fig. 6a). We then characterized the breast tumors according to the four major molecular subgroups, i.e., luminal A (*n* = 216), luminal B (*n* = 121), ERBB2 (*n* = 44), and triple-negative tumors (*n* = 67) [36]. All subgroups of breast tumors showed an increased level of VOPP1 transcripts (*p* = 1.10^−3^, 1.10^−3^, 1.10^−4^, and 6.10^−3^, respectively) (Fig. 6a). Of note, the ERBB2 tumors exhibited slightly higher amounts of VOPP1 compared to the other subgroups. To confirm these findings, we interrogated the Oncomine database for VOPP1 expression in several additional breast cancer datasets and obtained similar results (Additional file 4: Figure S3A-B).

In light of the *VOPP1* gene location within the 7p11.2 chromosomal region, which contains *EGFR*, and frequent amplification in several malignancies, including basal-like breast cancers [26, 27], we investigated whether altered *VOPP1* expression was due to gene amplification. We performed a CGH array analysis of samples that exhibited the highest levels of *VOPP1* mRNA (Fig. 6a). We found that 7p11.2 amplification was observed only in the triple-negative tumors (2/2 cases), whereas luminal and HER2 tumors showed no genomic alteration (0/5) (Fig. 6a and Additional file 4: Figure S3C). Therefore, gene amplification was not the main mechanism leading to *VOPP1* overexpression in breast cancers.

Next, we examined VOPP1 protein expression in a series of 24 human breast tumors. In all cases, adjacent non-cancerous tissue revealed weak staining for VOPP1 in the luminal and myoepithelial layers of the mammary glands. The VOPP1 protein showed a higher expression in the blood vessels (Fig. 6b). In accordance with our qRT-PCR results, breast tumors showed increased expression of *VOPP1* protein (Fig. 6c, *p* = 0.0002) and this higher expression was not linked to any specific breast tumor subtype (Additional file 5: Figure S4A-B). Notably, VOPP1 staining showed a predominant granular cytoplasmic distribution reminiscent of the vesicular structures observed in vitro (Fig. 6b and Additional file 5: Figure S4B).

To evaluate the prognostic value of the *VOPP1* expression on breast cancer patients’ survival, we performed a univariate analysis. As shown by the Kaplan-Meier curve, among the 448 tested patients, those having tumors with high *VOPP1* mRNA expression tended to have reduced metastasis-free survival compared to those expressing lower *VOPP1* mRNA levels, even though the difference was not statistically significant (Fig. 6d, the first graph on the left).

We then reasoned that the potential contribution of *VOPP1* expression in the unfavorable breast cancer clinical outcome might be due to its inhibitory effect on WWOX tumor suppressor activity. In this case, we expect that the impact of high *VOPP1* expression could be more relevant in tumors not inactivated for WWOX expression. Since it has previously been demonstrated that WWOX expression positively correlates with expression of hormone receptors [37–39], we thus performed a log-rank test in the subgroups of luminal tumors (the hormone receptor-positive tumors) and the luminal B subtype that are those with a higher risk of metastasis.

Interestingly, high *VOPP1* mRNA expression was significantly associated with an increased risk of metastasis in the subgroups of luminal tumors (log-rank, *p* = 0.003) and luminal B tumors (log-rank, *p* = 0.005). In accordance with our findings, the analysis of independent datasets showed a significantly worse prognosis in luminal B tumors (Additional file 5: Figure S4C). After adjusting for standard clinicopathological prognostic factors, *VOPP1* expression remained an independent prognostic factor in luminal B breast tumors (*p* = 0.003; Additional file 6: Table S2).

### Clinical relevance of the VOPP1-WWOX interaction in breast cancer

**Fig. 7:**
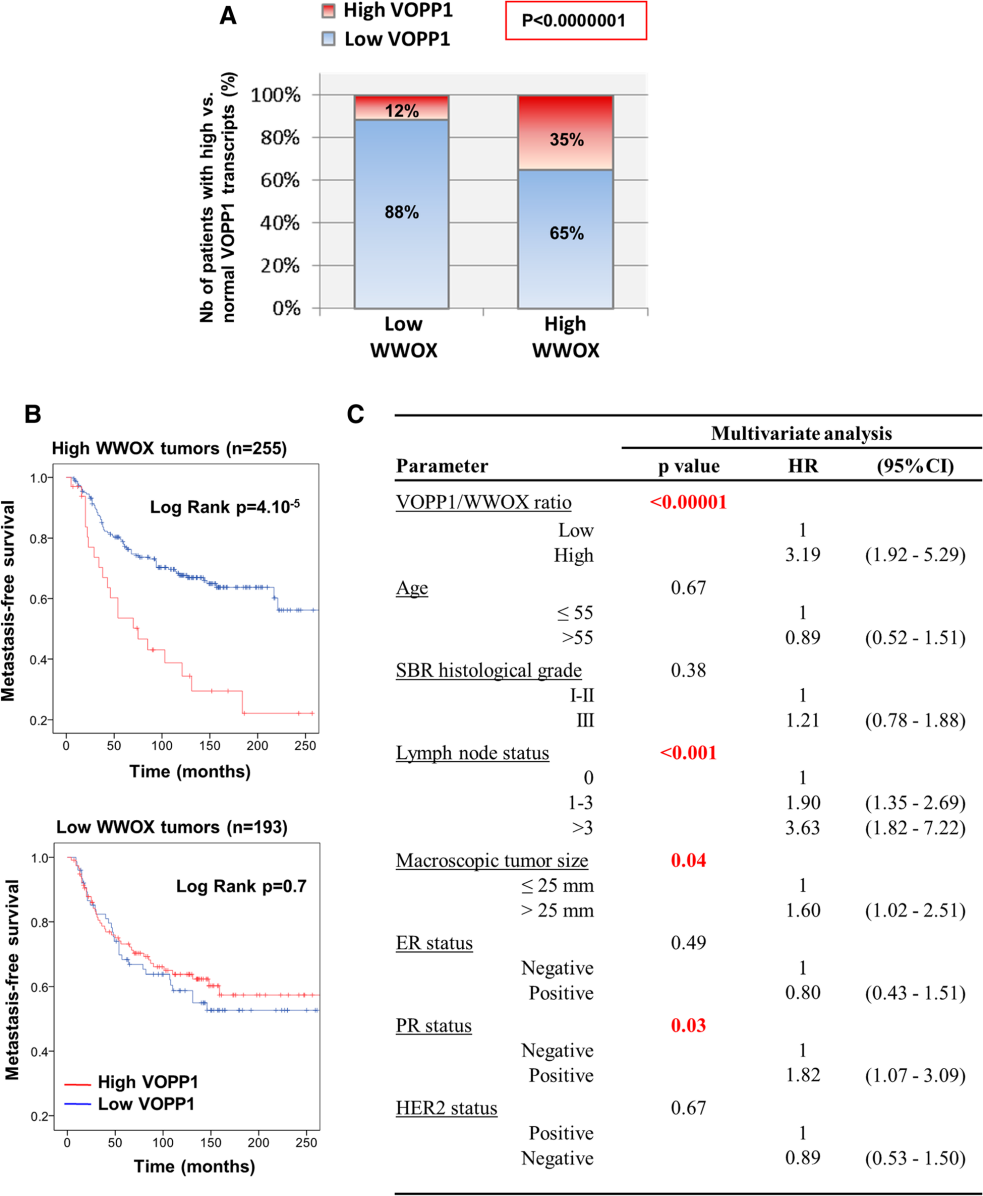
Concomitant expression of WWOX and VOPP1 in human breast tumors. **a** The 448 breast cancer patients were classified into two groups (low WWOX (< 0.6), *n* = 193; and high WWOX (≥ 0.6), *n* = 255), and the proportion of tumors expressing high (≥ 2.5) or low levels (< 2.5) of VOPP1 was assessed in each group. Statistical significance was calculated by the chi-square test (**b**). Kaplan-Meier curves showing metastasis-free survival rates of patients with tumors expressing high (red line) vs. low (blue line) VOPP1/WWOX ratio in subpopulations of patients with low or high WWOX levels. **c** Multivariate Cox proportional hazards analysis showing the independence of the VOPP1/WWOX expression ratio in the prognosis of breast cancer patients retaining WWOX expression (HR, hazard ratio; CI, confidence interval)

We next examined the concomitant expression of *VOPP1* and *WWOX* in our series of breast tumors. In keeping with previous reports [37–39], *WWOX* expression correlated positively with expression of hormone receptors, and reduced *WWOX* mRNA levels tended to be associated with poor prognosis, ER/PR-negative tumors (Additional file 3: Table S1 and Additional file 5: Figure S4D). Most importantly, we observed a negative correlation between *WWOX* underexpression and *VOPP1* overexpression (Fig. 7a). Indeed, tumors that expressed decreased levels of *WWOX* mainly showed low *VOPP1* expression (88%), whereas tumors with high *WWOX* levels exhibited a higher proportion of *VOPP1* overexpression (12 vs. 35%), (*p* < 1.10^−7^).

Since *VOPP1* and *WWOX* have concomitant patterns of expression, we evaluated the presence of significant interaction between *VOPP1* and *WWOX* expression on breast cancer metastasis-free survival rates. Therefore, we considered a Cox regression model, which included both variables (*WWOX* and *VOPP1* expression) and an interaction term (VOPP1*WWOX). Whereas *VOPP1* and *WWOX* had non-significant *p* values related to the outcome, the hazard ratio associated with the interaction term was estimated to be equal to 3.41 and was statistically significant (*p* = 0.02) indicating a substantial interaction of both variables in the patient metastasis-free survival (Additional file 7: Table S3).

To evaluate whether more discrete variations of *VOPP1* vs. *WWOX* expressions could predict the risk of breast cancer metastasis with a higher efficacy, we tested the *VOPP1*/*WWOX* ratio in univariate and multivariate analyses. Indeed, we found that the high level of *VOPP1*/*WWOX* ratio decreased the survival of patients that retained WWOX expression (log-rank test, *p* = 4.10^−5^) but not of patients whose tumors had *WWOX* gene alterations (Fig. 7b). Moreover, interestingly, in a multivariate model (Fig. 7c), the *VOPP1*/*WWOX* ratio was a highly significant variable (*p* < 0.00001) with a hazard ratio of 3.2 (95% CI 1.9–5.3).

All these observations support strongly that overexpression of VOPP1 induces breast cancer by impairing the tumor suppressive activity of WWOX.

## Discussion

In this study, we defined VOPP1 as a new molecular partner and an inhibitor of the apoptotic function of WWOX in breast cancer cells. We demonstrated that VOPP1 potentiated cellular transformation and enhanced tumorigenesis in vivo. Moreover, we showed that VOPP1 was overexpressed in breast tumors; this overexpression was predominant in tumors that retained WWOX expression and was associated with reduced metastasis-free survival of patients with WWOX-positive tumors. These findings emphasize the importance of WWOX compartmentation in addition to WWOX loss in breast tumors and define *VOPP1* as a novel oncogene that promotes breast carcinogenesis.

VOPP1 protein function has not yet been extensively studied. Analysis of the protein sequence did not highlight any obvious functional domains, although it did reveal a transmembrane domain and a signal peptide as predicted by TMHMM2.0 and SignalIP programs. In addition, the protein harbors two endosome/lysosome targeting sequences, namely a YXXΦ motif (Φ is a hydrophobic amino acid) and a dileucine sequence, which suggest its function in the sorting of proteins to the endosome, Golgi apparatus, and lysosome [40]. Interestingly, the VOPP1 protein was not the only WWOX partner that has been shown to belong to the endolysosomal compartment. The E3 ubiquitin ligase ITCH and LITAF/SIMPLE proteins also localize to endolysosomes [41]. LITAF is a tumor suppressor that was previously reported to physically interact with ITCH and induce the relocation of ITCH to the lysosomes, likely interfering with ITCH function. Most importantly, ITCH mediated K63-linked polyubiquitination of WWOX, which led to its nuclear localization and enhanced DDR [24]. Furthermore, the WWOX-ITCH interaction rendered cells more sensitive to p73 pro-apoptotic function [20]. Altogether, these data suggest that WWOX interactions are finely orchestrated to efficiently control the suppression of tumorigenic signaling pathways including DNA repair and tumor cell apoptosis.

Independent groups previously characterized VOPP1 as an antiapoptotic protein [31, 32]. Their studies highlighted different underlying molecular pathways, depending on the experimental systems that were used. First, ectopic expression of VOPP1 was shown to prevent apoptosis by regulating NF-κB transcriptional activity in HeLa cells. The authors demonstrated that VOPP1 knockdown delayed the degradation of IκBα, which inhibited the NF-κB pathway. These results might be consistent with our findings as WWOX has been suggested to physically interact with IκBα protein [42]. However, although the role of WWOX in the NF-κB pathway has been described in the human T cell leukemia virus type I (HTLV-I)-mediated tumorigenesis, the functional relationship between WWOX and NF-κB in other malignancies remains to be determined [43].

Alternatively, other studies reported that loss of VOPP1 induced apoptosis via control of the intracellular redox state [31]. In line with these data, increased ROS levels were observed in WWOX-overexpressing larvae in a *Drosophila* model while *Wwox* mutants had consistently lower levels of ROS [44]. Importantly, a key role of WWOX in cell metabolism was strongly supported by the severe metabolic defects leading to the early postnatal death of WWOX-null mice [45]. The molecular mechanisms underlying these complex metabolic functions of WWOX are still under investigation.

Our results suggest that VOPP1 promotes cancer by inhibiting WWOX-dependent apoptosis; however, we cannot exclude the possibility that VOPP1 acts on cancer by impairing other WWOX functions. This is supported by different studies and observations suggesting that WWOX can act on cancer independently of apoptosis. Notably, many cell lines survive following stable transfection of WWOX; therefore, apoptosis would not be the only mechanism by which WWOX works as a tumor suppressor. Moreover, WWOX interacts with, and modulates the activity of, numerous proteins, such as RUNX2 and Dvl-2, involved in migration and invasion, two cancer-promoting cellular processes [22, 46, 47]. The involvement of VOPP1 in the regulation of these apoptosis-independent WWOX functions remains to be investigated.

Growing evidence indicates that *WWOX* is not a classical tumor suppressor. Unlike most tumor suppressor genes, the inactivation of only one WWOX allele was sufficient to trigger tumor formation [17]. This haploinsufficient phenotype was demonstrated in mice with targeted knockout of a single copy of *WWOX* or mice with WWOX hypomorphism [14–17]. These studies revealed an increased incidence of lung and breast tumors and B cell lymphomas. Similar to the PTEN tumor suppressor, differences in the types of neoplasias observed in these animal models might be due to the regulation of WWOX by various molecular mechanisms, including VOPP1-mediated relocation, which generates dosage-dependent WWOX functions in human cancers. Further investigations are still required to fully unravel this question.

Our data suggested that the involvement of the WWOX pathway in breast cancers might have been underestimated. Consistent with this possibility, by studying our series of 448 breast tumors, we found that the alteration of WWOX function, considered either by underexpression of *WWOX* or by overexpression of *VOPP1*, affects 81.69% (364/448) of breast tumors. Our findings may also shed new light on the potential involvement of the WWOX/VOPP1 axis in a broader range of human malignancies. Overexpression of *VOPP1* has been reported in several cancers such as a glioblastoma and head and neck, lung, gastric, and pancreatic carcinomas [28, 30], which are malignancies commonly affected by *WWOX* loss of expression [1]. Therefore, the altered expression of WWOX and aberrant localization should be more precisely scrutinized in these cancers; further studies on this pathway may provide a better assessment of the WWOX/VOPP1 axis in human cancers.

## Conclusions

Our findings shed new light on WWOX localization and its role in apoptosis and breast tumorigenesis. Whereas a therapeutic approach aimed at restoring WWOX loss of function is not straightforward, our findings open avenues for the development of new therapies targeting this pathway by affecting the VOPP1 oncogene in a broad range of human malignancies.

## Methods

### DNA constructs

The Myc-WWOX and Myc-WWOX_Y33R_ expression vectors were kindly provided by Dr. R.I. Aqeilan. Full-length *WWOX* cDNA was cloned into a Myc-tagged pCMV vector (BD Clontech, Palo Alto, CA) by using standard protocols. pCMV-Myc-WWOX_Y33R_ plasmid (Myc-WWOX_Y33R_) was obtained by site-directed PCR mutagenesis (Stratagene, La Jolla, CA), according to the manufacturer’s instruction [23]. HA-p73α was kindly provided by Dr. G. Melino. p73 cDNA was synthetized by RT-PCR using total RNA extracted from the SH-Sy5y cell line cell. p73 cDNA was sequenced and cloned in-frame with a hemagglutinin (HA) tag into pcDNA3-HA using the NheI and NotI unique restriction sites. The pCDNA3-HA plasmid was constructed by insertion of a BglII/BamHI fragment from pActII (Clontech) into the BamHI site of pCDNA3 (Invitrogen, Carlsbad, CA) [48, 49]. *VOPP1* cDNA (519 bp, NM_030796) was amplified by RT-PCR from human tissues (Clontech) expressing the highest levels of VOPP1 RNA (normal kidney, breast, and placenta), as determined in silico by means of the Serial Analysis of Gene Expression (SAGE) program. The PCR product was cloned into the expression vectors p3xflag-CMV-7.1 (Sigma-Aldrich, Saint-Louis, Missouri) (Flag-VOPP1) and pIREShyg3 (Clontech) by using appropriate primers. Site-directed mutagenesis was performed to independently modify the tyrosine residue to alanine in each PPxY motif of the VOPP1 construct according to the manufacturer’s instructions (Stratagene, La Jolla, CA). The tyrosine residue of each PPxY motif was mutated to alanine, generating three mutants: VOPP1_Y119A_, VOPP1_Y157A_, and VOPP1_Y165A_. All constructs were verified by DNA sequencing.

### Cell lines and transfections

All cell lines were purchased from the American Type Culture Collection (ATCC, Manassas, VA) and authenticated every 20 passages using the GenePrint 10 System kit (Promega, Madison, WI). Cells were grown in Dulbecco’ s minimal essential medium (DMEM) or minimal essential medium (MEM), supplemented with 10% fetal bovine serum (Invitrogen, Carlsbad, CA) and 1% antibiotics (50 μg/ml penicillin, 50 μg/ml streptomycin, 100 μg/ml neomycin; Invitrogen), and maintained at 37 °C with 5% CO_2_. Transfections were achieved using Lipofectamine according to the manufacturer’s instructions (Invitrogen). To generate NIH3T3 cells stably expressing VOPP1, cells were cultured for 3 weeks in a selection medium containing hygromycin (100 μg/ml). Dozens of hygromycin-resistant clones were then picked and expanded.

### Knockdown experiments, proliferation, and apoptosis assays

MDA-MB-468 cells were transfected using the HipPerFect Reagent following the manufacturer’s protocol with either the siRNA-negative control or siRNA-VOPP1 and/or siRNA-*WWOX* (#1027281, #SI03144967, and #SI02777775) (Qiagen, Valencia, CA). For the proliferation assay, MDA-MB-468 cells were seeded in 96-well plates and transfected with control or *VOPP1* siRNAs at day 0 and day 3. Cell number was measured using the MTS assay according to the instructions of the manufacturer (Promega). The absorbance at 490 nm was performed on a 96-well microplate reader (Dynatech Laboratories MRX, Chantilly, VA). Cell death was evaluated using the FITC Annexin V kit (BD Biosciences, San Jose, CA). Briefly, cells were washed in PBS with EDTA (1 mM) and resuspended in Annexin V binding buffer (10^6^ cells/ml). Samples were incubated with Annexin V-FITC antibody for 15 min in the dark and propidium iodide was added. Data were acquired on a Becton Dickinson LSR II flow cytometer and analyzed using FlowJO V10 software.

### Immunofluorescence microscopy, 3D deconvolution, and image analysis

Cells plated on glass coverslips were fixed (PBS, 4% paraformaldehyde, 10 min), permeabilized (PBS, 0.1% Tween-20, 10 min), and incubated with PBS containing 5% goat serum and appropriate antibodies. Cells were then stained with DAPI and examined with a fluorescence microscope (Eclipse Ti-S Nikon, Melville, NY). Images were acquired with a wide-field Eclipse 90i Upright Microscope (Nikon) using a × 100 Plan Apo VC 1.4 oil immersion objective and a highly sensitive cooled interlined charge-coupled device (CCD) camera (Roper CoolSnap HQ2). A z-dimension series of images was taken every 0.2 μm via a piezoelectric motor (LVDT; Physik Instrument), and images were deconvoluted [50]. Acquisition, analyses, and quantifications were performed using the MetaMorph Imaging System (Universal Imaging Corp.) and ImageJ software.

### Antibodies, western blotting, and immunoprecipitation

Anti-VOPP1 polyclonal antibody was produced by inoculating rabbits with two distinct VOPP1 peptides corresponding to amino acids 101-116 (TRQPPNPGPGTQQPGP) and amino acids 137-151 (AFQVPPNSPQGSVAC) (Eurogentec, Seraing, Belgium). The other antibodies used are as follows: anti-WWOX [22]; anti-WWOX (ref.: sc-20528, Santa Cruz, Dallas, TX, RRID:AB_2216503); anti-VOPP1 (ref.: 12611-1AP, Proteintech, Chicago, IL); anti-GM130 (re.: 610822, BD Biosciences, San Jose, CA, RRID:AB_398141) and anti-EEA1 (ref.: 610457, BD Biosciences, San Jose, CA RRID:AB_397830); anti-LAMP2 (ref.: ab25631, Abcam, Cambridge, MA, RRID:AB_470709); anti-cleaved PARP (ref.: 9541, Cell Signaling, Danvers, MA, RRID:AB_331426); anti-Flag (ref.: F3165, Sigma-Aldrich St Louis, MO, RRID:AB_259529); anti-HA (ref.: MMS-101P, Covance, Indianapolis, IN, RRID:AB_2314672); anti-Myc (ref.: 631206, Clontech Palo Alto, CA); secondary Alexa fluor-conjugated antibodies (ref.: A-11011, RRID:AB_143157, ref.: A-11031, RRID:AB_144696, ref.: A-11034, RRID:AB_2576217, ref.: 11068, RRID:AB_2534112, ref.: 11039, RRID:AB_2534096, Invitrogen Thermo Fisher Scientific Rockford, IL); IgG-dylight 650-conjugated (ref.: A90-516D5, Bethyl Laboratories, Montgomery, TX, RRID:AB_10631368), and HRP-linked antibodies (ref.: 111-035-003, RRDI: AB_2313567, ref.: 115-035-062, RRDI:AB_2338504, ref.: 705-035-003, RRDI:AB_2340390, Jackson ImmunoResearch, West Grove, Pennsylvania). Western blotting and co-immunoprecipitation methods were previously described [51]. For endogenous WWOX-VOPP1 complexes, cell extracts containing 800 μg of total proteins were subjected to direct immunoprecipitation with the anti-VOPP1 antibody.

### Colony formation in soft agar and tumorigenicity assay

3.10^4^ NIH3T3 cells, stably expressing VOPP1 (3T3-VOPP1) or empty vector (ctrl-VOPP1), were mixed with melted 0.35% agarose in DMEM medium and seeded on top of a 0.7% agarose base layer containing the same medium. Culture medium was changed twice per week for 4 weeks, and cells were observed with an optical microscope. 2.10^6^ 3T3-VOPP1 or ctrl-VOPP1 cells were injected subcutaneously into the flanks of 7-week-old immunodeficient mice (CB17 SCID, Charles Rivers Laboratories). In a first experiment (Fig. 5e), tumor dimensions were measured twice weekly until the sacrifice at day 35. A second experiment was carried out and followed for a longer time period (Fig. 5f). Mice were sacrificed when tumors reached a volume of approximately 2 cm^3^ (volume = *a* × *b*^2^/2; *a* and *b* are the two registered perpendicular diameters, with *a* > *b*); otherwise, they were monitored for 6 months (control mice). Animal care and use for this study were performed in accordance with the recommendations of the European Community (2010/63/UE) for the care and use of laboratory animals. Experimental procedures were specifically approved by the ethics committee of the Institut Curie CEEA-IC #118 (approval: 2016-014) in compliance with international guidelines.

### Breast tumor samples and expression analyses

Normal breast tissues from ten women undergoing cosmetic breast surgery were used as a source of normal RNA. Four hundred forty-eight primary breast tumor samples were retrospectively collected from patients undergoing surgery at the Institut Curie-Hospital Huguenin. The sample series was specifically selected to encompass the various stages of breast cancer progression and the tumors’ molecular subtypes, defined as previously described [36]. The median follow-up of the patients was 101.7 months (range = 4.3–250 months). The study was performed in accordance with the French Bioethics Law 2004–800 and the French National Institute of Cancer (INCa) Ethics Charter, and after approval by the Institut Curie review board and ethics committee (Comité de Pilotage du Groupe Sein), which waived the need for written informed consent from the participants. Women were informed of the research use of their tissues and did not declare any opposition to the research. Total RNA extraction, cDNA synthesis, and PCR reaction conditions have been described in detail elsewhere [52]. *WWOX* or *VOPP1* mRNA levels were calculated using the ΔΔ*C*t method and normalized to the TATA-box-binding protein transcript expression levels. The primers sequences were as follows: *VOPP1-for*: 5′-GGCTGTGGTACTTCTGGTTCCTT-3′, *VOPP1-rev*: 5′-GTGTAGGACACATTGAAGGCTGG-3′, *WWOX-for*: 5′-CTGGGTTTACTACGCCAATCACA-3′, and *WWOX-rev*: 5′-GCAAATCTCCTGCCACTCGTT-3′. For the immunohistochemical study, paraffin-embedded sections of human tumors were prepared as described [53]. Briefly, human biopsy specimens of 24 primary breast tumors were deparaffinized, treated with 3% H_2_O_2_, and incubated with anti-VOPP1 antibodies (Proteintech). The staining signals were revealed with the Dako REAL Detection System, Peroxidase/AEC kit. The slides were counterstained with Mayer’s hematoxylin.

### CGH array

Tumors with high levels of *VOPP1* transcripts were subjected to DNA extraction. The quality of tumor DNAs was verified using a Qubit dsDNA BR Assay Kit (Thermo Fisher Scientific). Tumor DNAs and reference DNA provided in the Agilent Kit were labeled, purified, and co-hybridized in equal quantity to the Agilent Microarrays, for 21–24 h. Arrays were washed and scanned on a SureScanMicroarray Scanner according to the manufacturer’s protocols (Agilent). Images were acquired using the CytoScan Software V.2.7 (Thermo Fisher Scientific) and analyzed on CytoGenomics Software V.2.7 (Agilent).

### Statistical analysis

Statistical calculations were performed using PASW Statistics (version 18.0; SPSS Inc.). To determine whether *VOPP1* expression and the *VOPP1*/*WWOX* ratio were associated with patient clinical outcome, survival distributions were estimated by the Kaplan-Meier method, and the significance of differences between survival rates was ascertained using the log-rank test. The classification of tumors in “high VOPP1” or “reduced WWOX” vs. “normal groups” in Fig. 7 were obtained by using a threshold of < 0.6 for reduced WWOX levels and ≥ 2.5 for high VOPP1 levels. Multivariate analysis using Cox proportional hazards model was used to assess the independent contribution of each variable to metastasis-free survival. The Cox proportional hazards assumption was graphically checked by plotting a log-cumulative hazard rate for each variable, and we did not find statistical evidence for violation of this assumption in any of the regression models.

## Additional files

Additional file 1: **Figure S1.** VOPP1 relocates WWOX into a cytoplasmic vesicular compartment. (A, B) Localization of exogenous WWOX in the presence of exogenous VOPP1 or VOPP1_Y165A_ in Hela cells. Cells were transfected with WWOX and VOPP1 or VOPP1_Y165A_ expression vectors as indicated. At 24 h, cells were fixed, permeabilized, and immunostained with anti-WWOX and anti-VOPP1 antibodies, and with anti-LAMP2 (A) or anti-GM130 (B) antibodies. Cells were then counterstained with DAPI and imaged using a fluorescence microscope (original magnification: X100). (TIF 1756 kb)

Additional file 2: **Figure S2**. Effect of the overexpression of WWOX and VOPP1 on cell death. (A) A549 cells were transfected with empty vector (Ctrl), WWOX, and VOPP1 expression constructs as indicated. Three days later, cells were collected and the percentage of apoptotic cells was evaluated by flow cytometry analysis. (B) Numbers of apoptotic cells are expressed as mean ± SEM of results from three different experiments. Statistical analysis was performed by Student *t* test (*p < 0.05; **p < 0.01). (TIF 1171 kb)

Additional file 3: **Table S1.** Correlation between *WWOX* and *VOPP1* mRNA expression and the clinocopathological parameters in 448 breast cancers. (TIF 401 kb)

Additional file 4: **Figure S3.** VOPP1 expression and gene amplification in human breast tumors. (A) Box plots of *VOPP1* expression levels (normalized expression units) in independent microarray studies obtained from the Oncomine database (https://www.oncomine.org). Differences between normal and cancerous tissues are shown for four breast cancer datasets (TCGA, *p* < 10^−18^; Curtis, *p* < 10^−54^; Gluck, *p* = .015; Student’s *t* test). (B) *VOPP1* expression in the four molecular subtypes in two independent cohorts “EMC” (*n* = 344; GSE2034 and GSE5327), and “NKI” (*n* = 295; Netherlands Cancer Institute). C) The CGH profiles showing VOPP1 chromosomal region are detailed for the two tumors highlighted in Fig. 6. (TIF 631 kb)

Additional file 5: **Figure S4.** VOPP1 protein expression in human breast tumors and correlation with clinical outcome. (A) Quantification of the immunohistochemical results shown in Fig. 6c, with respect to the breast cancer subtypes. (B) Representative images of an immunohistochemical analysis of VOPP1 protein expression in normal breast tissues and breast tumors. (C) Kaplan-Meier curves showing metastasis-free survival rates of patients with tumors expressing high (red line) vs. low (blue line) levels of VOPP1 mRNA in “EMC” and “NKI” cohorts or luminal B subsets of the cohorts. (D) Kaplan-Meier curves showing metastasis-free survival rates of patients with tumors expressing high (red line) vs. low (blue line) levels of WWOX mRNA assessed by RT-PCR in breast tumors samples. (TIF 1827 kb)

Additional file 6: **Table S2.** Cox proportional hazard analysis for MFS in two independent cohorts of patients with luminal B tumors. (TIF 344 kb)

Additional file 7: **Table S3.** Cox proportional hazard analysis for MFS in the 448 breast tumors of Curie Cohort. (TIF 109 kb)
