# Supplementary figures and images for: VOPP1 promotes breast tumorigenesis by interacting with the tumor suppressor WWOX
Source: BMC Biol. 2018 Oct 2;16:109. doi: 10.1186/s12915-018-0576-6 (PMC6169085; doi:10.1186/s12915-018-0576-6)

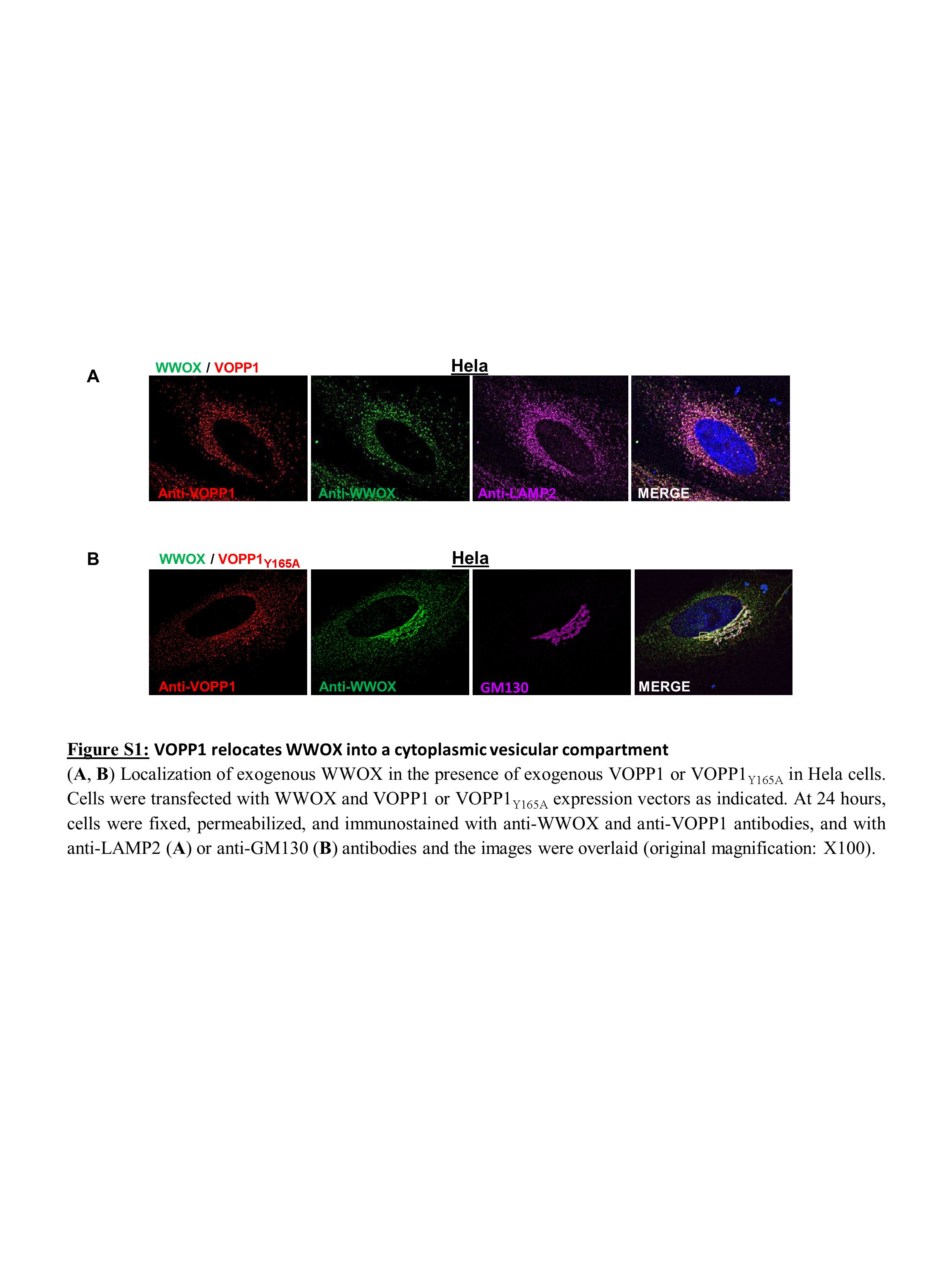

Supplement: Supplementary file 1 — Figure S1. VOPP1 relocates WWOX into a cytoplasmic vesicular compartment. (A, B) Localization of exogenous WWOX in the presence of exogenous VOPP1 or VOPP1Y165A in Hela cells. Cells were transfected with WWOX and VOPP1 or VOPP1Y165A expression vectors as indicated. At 24 h, cells were fixed, permeabilized, and immunostained with anti-WWOX and anti-VOPP1 antibodies, and with anti-LAMP2 (A) or anti-GM130 (B) antibodies. Cells were then counterstained with DAPI and imaged using a fluorescence microscope (original magnification: X100). (TIF 1756 kb) [file 12915_2018_576_MOESM1_ESM.tif]

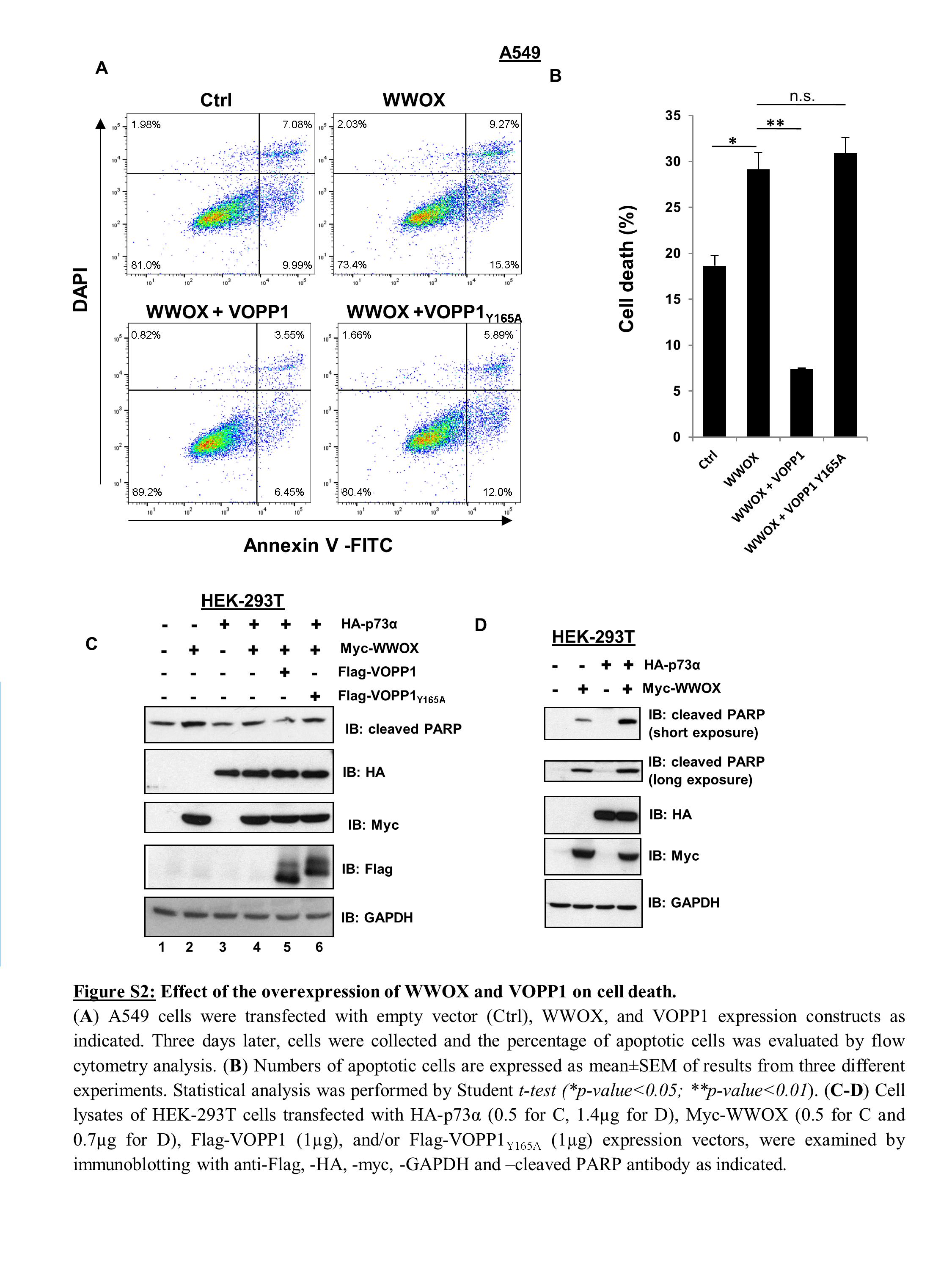

Supplement: Supplementary file 2 — Figure S2. Effect of the overexpression of WWOX and VOPP1 on cell death. (A) A549 cells were transfected with empty vector (Ctrl), WWOX, and VOPP1 expression constructs as indicated. Three days later, cells were collected and the percentage of apoptotic cells was evaluated by flow cytometry analysis. (B) Numbers of apoptotic cells are expressed as mean ± SEM of results from three different experiments. Statistical analysis was performed by Student t test (*p < 0.05; **p < 0.01). (TIF 1171 kb) [file 12915_2018_576_MOESM2_ESM.tif]

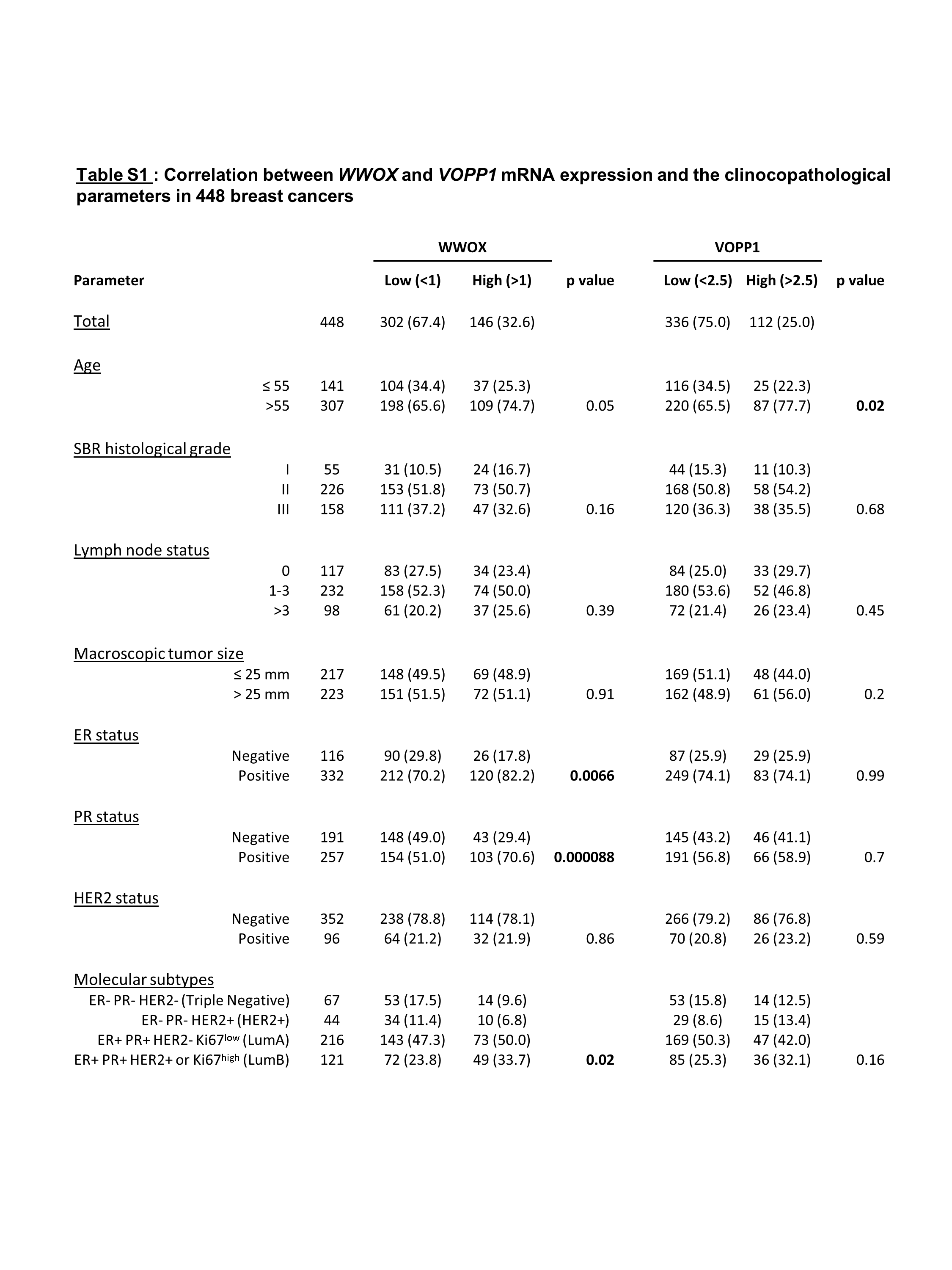

Supplement: Supplementary file 3 — Table S1. Correlation between WWOX and VOPP1 mRNA expression and the clinocopathological parameters in 448 breast cancers. (TIF 401 kb) [file 12915_2018_576_MOESM3_ESM.tif]

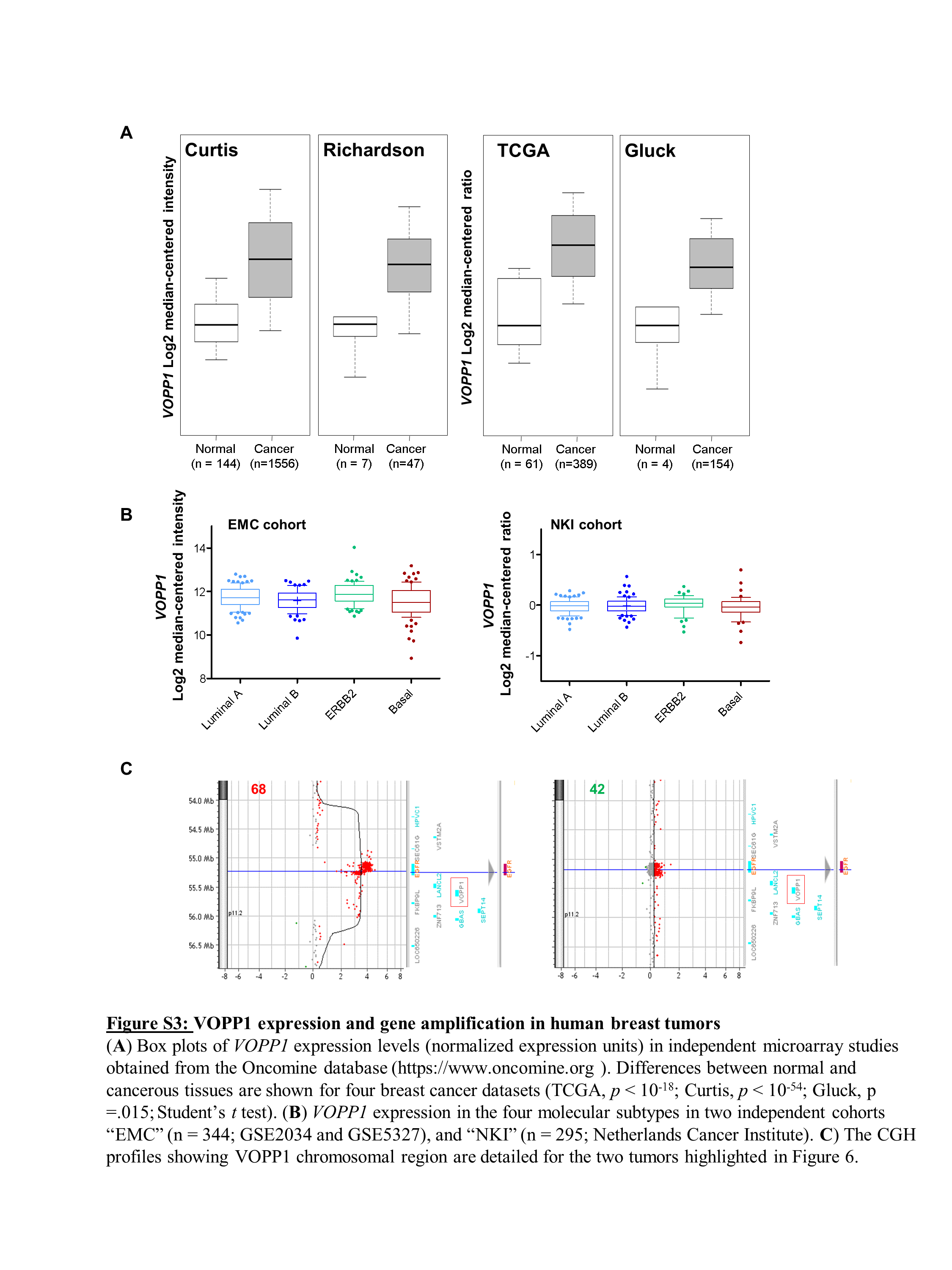

Supplement: Supplementary file 4 — Figure S3. VOPP1 expression and gene amplification in human breast tumors. (A) Box plots of VOPP1 expression levels (normalized expression units) in independent microarray studies obtained from the Oncomine database (https://www.oncomine.org). Differences between normal and cancerous tissues are shown for four breast cancer datasets (TCGA, p < 10−18; Curtis, p < 10−54; Gluck, p = .015; Student’s t test). (B) VOPP1 expression in the four molecular subtypes in two independent cohorts “EMC” (n = 344; GSE2034 and GSE5327), and “NKI” (n = 295; Netherlands Cancer Institute). C) The CGH profiles showing VOPP1 chromosomal region are detailed for the two tumors highlighted in Fig. 6. (TIF 631 kb) [file 12915_2018_576_MOESM4_ESM.tif]

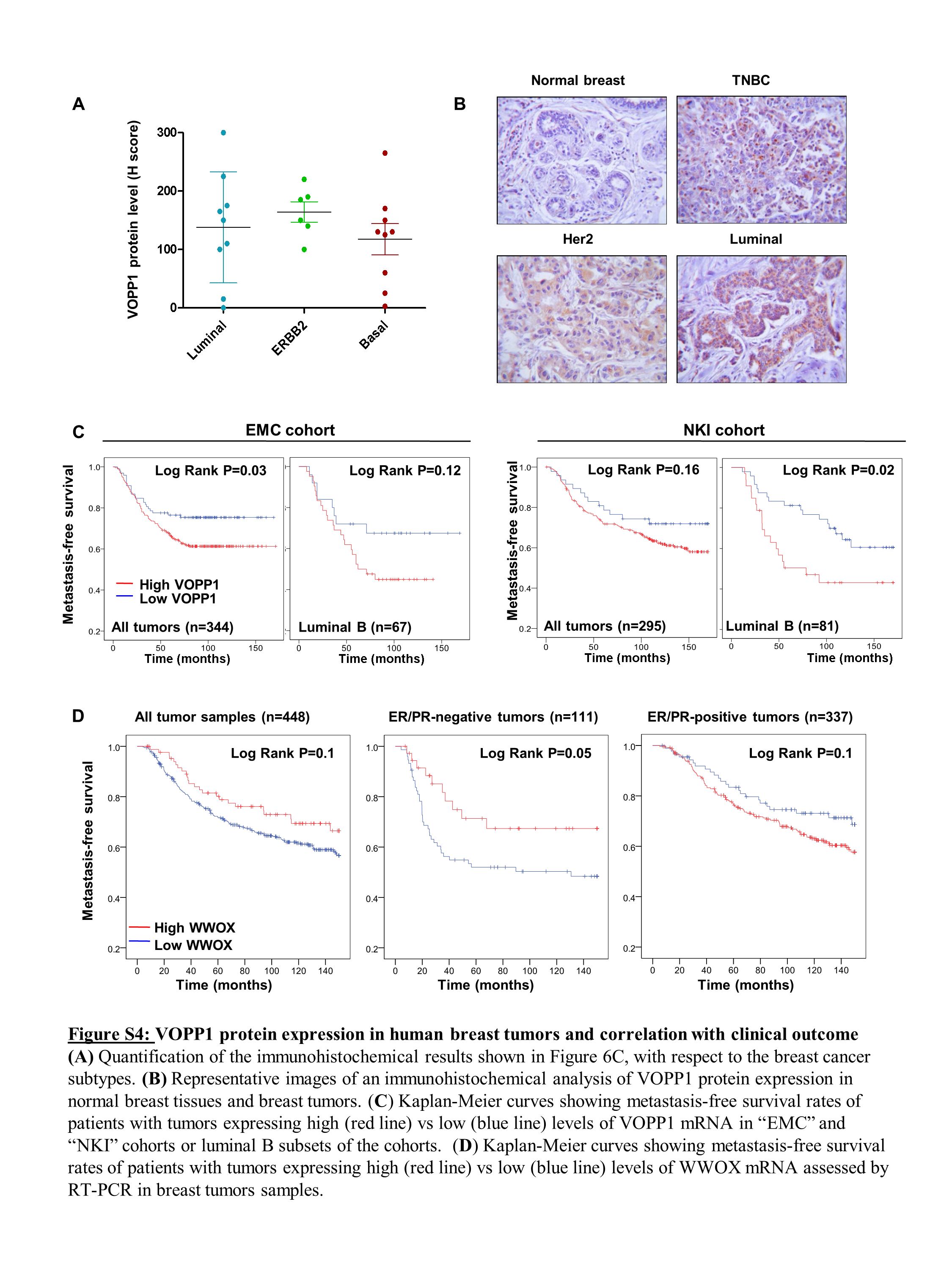

Supplement: Supplementary file 5 — Figure S4. VOPP1 protein expression in human breast tumors and correlation with clinical outcome. (A) Quantification of the immunohistochemical results shown in Fig. 6c, with respect to the breast cancer subtypes. (B) Representative images of an immunohistochemical analysis of VOPP1 protein expression in normal breast tissues and breast tumors. (C) Kaplan-Meier curves showing metastasis-free survival rates of patients with tumors expressing high (red line) vs. low (blue line) levels of VOPP1 mRNA in “EMC” and “NKI” cohorts or luminal B subsets of the cohorts. (D) Kaplan-Meier curves showing metastasis-free survival rates of patients with tumors expressing high (red line) vs. low (blue line) levels of WWOX mRNA assessed by RT-PCR in breast tumors samples. (TIF 1827 kb) [file 12915_2018_576_MOESM5_ESM.tif]

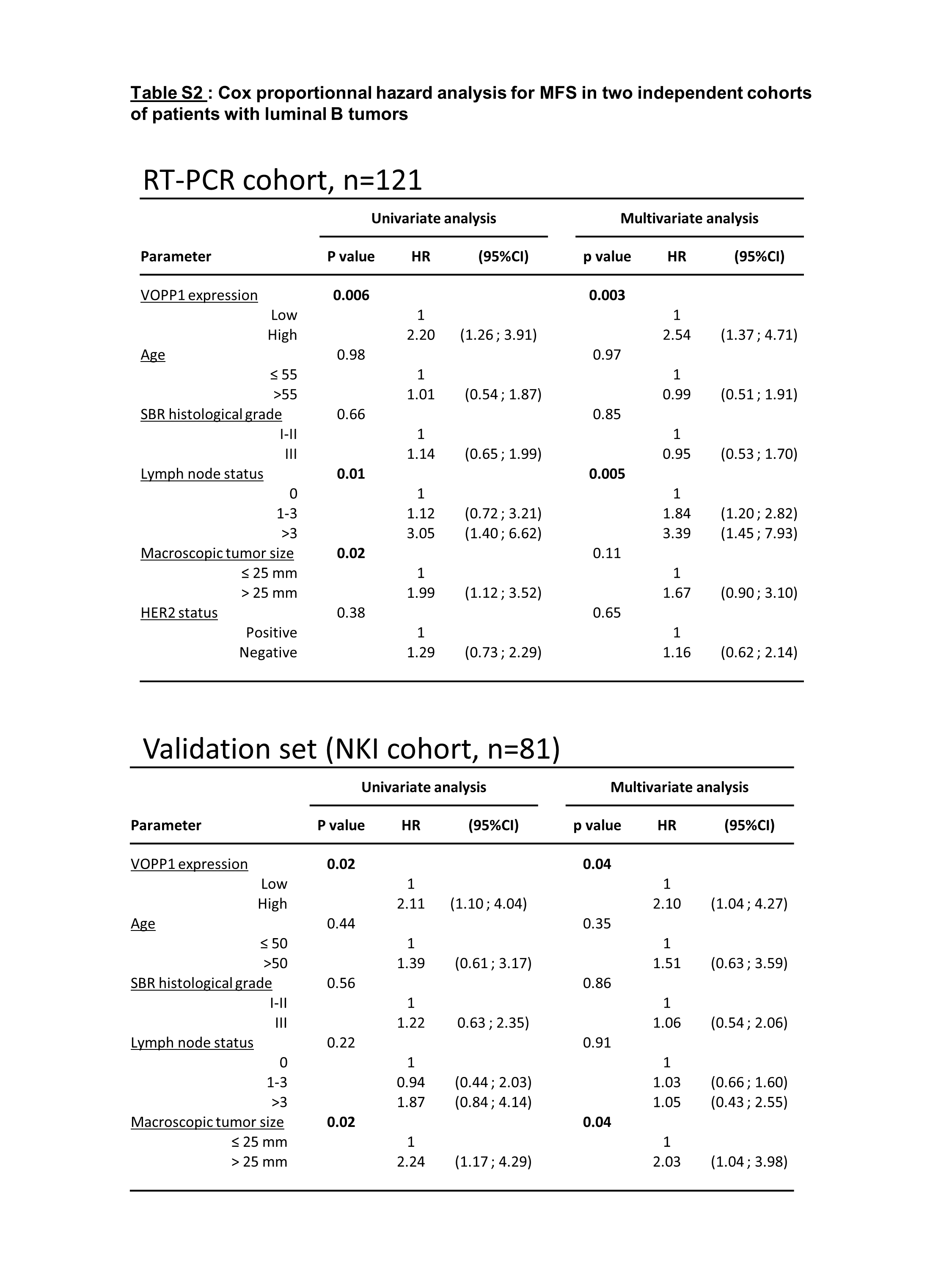

Supplement: Supplementary file 6 — Table S2. Cox proportional hazard analysis for MFS in two independent cohorts of patients with luminal B tumors. (TIF 344 kb) [file 12915_2018_576_MOESM6_ESM.tif]

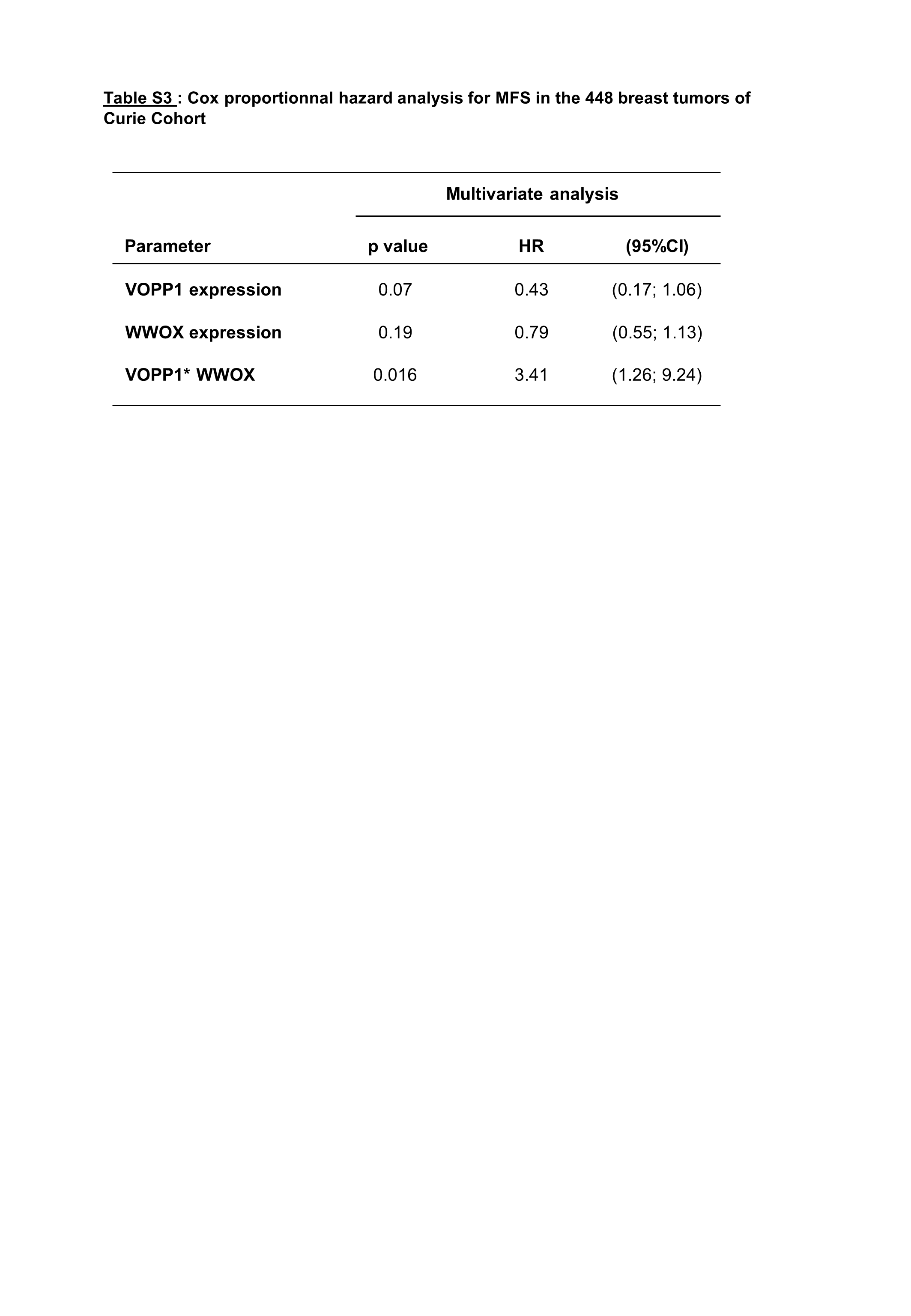

Supplement: Supplementary file 7 — Table S3. Cox proportional hazard analysis for MFS in the 448 breast tumors of Curie Cohort. (TIF 109 kb) [file 12915_2018_576_MOESM7_ESM.tif]
